# Induction of Callogenesis, Organogenesis, and Embryogenesis in Non-Meristematic Explants of Bleeding Heart and Evaluation of Chemical Diversity of Key Metabolites from Callus

**DOI:** 10.3390/ijms21165826

**Published:** 2020-08-13

**Authors:** Dariusz Kulus, Alicja Tymoszuk

**Affiliations:** Laboratory of Ornamental Plants and Vegetable Crops, Faculty of Agriculture and Biotechnology, UTP University of Science and Technology in Bydgoszcz, Bernardyńska 6, 85-029 Bydgoszcz, Poland; alicja.tymoszuk@utp.edu.pl

**Keywords:** anthocyanins, auxins, carotenoids, chlorophylls, cytokinins, polyphenols, spectral assay

## Abstract

*Lamprocapnos spectabilis* (L.) Fukuhara is a perennial plant species valued in the horticultural, cosmetic, and pharmaceutical markets. To date, however, there were no studies on tissue culture systems in this species when adjusted from non-meristematic explants. The aim of this study is to induce callogenesis, organogenesis, and somatic embryogenesis in non-meristematic explants of *Lamprocapnos spectabilis* ‘Alba’ cultured in various media and to analyze the chemical diversity of the produced callus. Leaf, petiole, and internode explants were cultured on the modified Murashige and Skoog (MS) medium fortified with various combinations and concentrations of 6-benzyladenine (BA), indole-3-acetic acid (IAA), 1-naphthaleneacetic acid (NAA), 2,4-dichlorphenoxyacetic acid (2,4-D), and picloram (PIC). After 10 weeks of culturing, the morphogenetic response of explants was evaluated and the concentration of chlorophylls, carotenoids, anthocyanins, and polyphenols in callus was analyzed. There was no influence of explant type on the callogenesis efficiency (62.1–65.3%). The highest fresh weight of callus was produced on leaf explants in the presence of 2,4-D or PIC. In contrast, the highest share of dry weight was found in internode-derived calli and cultured on IAA-supplemented medium (up to 30.8%). Only 2.5% of all explants regenerated adventitious shoots, while rhizogenesis was reported in 4.5% of explants. Somatic embryos were produced indirectly by 0% to 100% of explants, depending on the culture medium and explant type. The highest mean number of embryos (11.4 per explant) was found on petioles cultured in the MS medium with 0.5 mg·L^−1^ BA and 1.0 mg·L^−1^ PIC. Calli cultured in media with NAA usually contained a higher content of primary and secondary metabolites. There was also a significant impact of explant type on the content of anthocyanins, polyphenols, and carotenoids in callus. Further studies should focus on the elicitation of metabolites production in callus culture systems of the bleeding heart.

## 1. Introduction

*Lamprocapnos spectabilis* (L.) Fukuhara (syn. *Dicentra spectabilis* (L.) Lem.) or bleeding heart is a perennial plant species, originating from Asia. It is a member of a small botanical family known as Fumariaceae, which is closely related to Papaveraceae. Due to its spectacular flowers, the species is commonly used as both an indoor and outdoor/landscape plant [1]. There is also lots of information on the health-stimulating properties of extracts derived from the bleeding heart. Its roots have been used in Asian folk medicine for treating pus and paralysis [2]. Strong anti-aging effects, against UV-induced skin photoaging, of *L. spectabilis* extracts, were observed even at a low (0.1% *w/v*) concentration [3]. Studies conducted by McNulty et al. [4] revealed the presence of antidepressants and apoptosis-inducing lactones (butenolides, menisdaurilide, and aquilegloide), which are useful in the elimination of human tumor cell lines at 10 µM concentration. Moreover, isoquinoline alkaloids present in bleeding heart can be applied in treating inflammation [5] and several other conditions [6]. The species is also a source of natural fungicides and antibacterial alkaloids useful in eliminating methicillin-resistant strains of *Staphylococcus aureus* F. J. Rosenbach [7]. Therefore, more attention should be focused on that species and the possible acquisition of its valuable compounds in vitro.

Similar to other plant species, the bleeding heart was introduced to in vitro culture conditions for large scale reproduction. Micropropagation techniques used with horticultural crops include shoot culture (i.e., induction of axillary shoots from the existing meristems) and regeneration via adventitious organogenesis, somatic embryogenesis (SE), and protocorm-like-bodies. Regeneration may occur either directly, from explant cuttings to shoots, or indirectly, with passage through a callus phase. Lee and Lee [8] reported indirect SE from seeds of the bleeding heart cultured in medium supplemented with various concentrations of 2,4-dichlorophenoxyacetic acid (2,4-D). About 64.2% of the somatic embryos converted to rooted plantlets with a 46% survival rate when acclimatized ex vitro. Kulus [9] developed a micropropagation protocol via axillary bud activation in different culture media for two commercial cultivars of *L. spectabilis* and achieved up to 3.3 new shoots from a single-node explant. On the other hand, there are no reports on adventitious organogenesis induced from non-meristematic explants in this species.

Due to the high accessibility and totipotency of plant cells, non-meristematic organs such as leaf blades, leaf petioles, and internodes are a good explant source for micropropagation, which is useful in breeding [10]. By placing them in a properly optimized medium, a change in their native developmental program takes place and adventitious embryos, shoots, and/or roots are produced. During this process, specialized cells lose their differentiated character and rejuvenate into a ‘stem cell-like state’ that confers a pluripotential [11]. In vitro culture conditions, especially medium supplementation with plant growth regulators (PGRs) and explant type, play an essential role in this process [12]. 

PGRs were first described in the mid-20th century by Skoog, Miller, and Tsui (reviewed by Thorpe [13]). Among the most commonly applied types of growth regulators, one can find auxins (AXs) and cytokinins (CKs). Those growth regulators are complementary and generally have numerous opposite effects. Auxins were the first PGRs described and are essential for cell growth, which affects both cell division and cellular expansion. Under in vitro conditions, AXs usually stimulate the development of roots and callus. They are also often used to initiate SE [14]. Among the most popular AXs added into culture media, one can find 2,4-D, indole-3-acetic acid (IAA), indole-3-butyric acid (IBA), 1-naphthaleneacetic acid (NAA), and picloram (PIC). Cytokinins, on the other hand, promote cell division and differentiation. Under in vitro conditions, they usually stimulate axillary/adventitious bud activation and maturation of somatic embryos [15]. The most commonly used CKs are 6-benzyladenine (BA), kinetin (KIN), thidiazuron (TDZ), and zeatin (ZEA). However, the final effect of PGRs is a species-dependent, cultivar-dependent, and even explant-dependent issue [16].

Besides biomass formation, PGRs affect the plant metabolism and synthesis of chemical compounds in which some are important for the industry. Plant tissue culture systems are used in the acquisition of secondary metabolites used as pharmaceuticals, agrochemicals, flavors, fragrances, coloring agents, biopesticides, and food additives [17]. Callus cultures emerged as a particularly useful system in achieving this goal [18]. By following specific strategies, it is possible to produce significant amounts of biomass with an increase in the accumulation of chemical compounds [17]. The common plant species, where callus cultures were used for the overproduction of secondary metabolites, are *Centella asiatica* L., *Stevia rebaudiana* Bertoni, *Vaccinium myrtillus* L., *Hypericum perforatum* L. var. *angustifolium*, *Maackia amurensis* Rupr., *Sophora flavescens* Aiton, *Ononis arvensis* L., and *Maclura pomifera* Raf. [19]. Production of metabolites in vitro can be more reliable, simpler, and more predictable when compared to uncontrolled in vivo conditions [18]. Unfortunately, there are no profound reports on the chemical composition of callus produced from bleeding heart explants or the impact of tissue culture conditions on this parameter. Such studies are necessary to improve the commercial use of *L. spectabilis*.

The aim of this study was to induce callogenesis, organogenesis, and somatic embryogenesis in non-meristematic explants of *Lamprocapnos spectabilis* ‘Alba’ cultured in various media in order to analyze the chemical diversity of the produced callus.

## 2. Results

### 2.1. Morphogenetic Response of Explants—Callogenesis and Organogenesis 

Callogenesis began in the third week of the culture. Varied effectiveness in callus induction was observed, depending on the experimental object (Table 1, Supplementary Figure S1A). No callogenesis was reported in the PGR-free control medium (M0). Media M1 (0.5 mg·L^−1^ BA + 1.0 mg·L^−1^ IAA) and M4 (1.0 mg·L^−1^ BA + 2.0 mg·L^−1^ IAA) were less effective in stimulating callus development (59.3–66.7% of responding explants) than other PGR combinations (70.4–100%). On the other hand, there was no influence of explant type on this parameter. The callogenesis is reported in 62.1%, 62.4%, and 65.3% of internode, petiole, and whole-leaf explants, respectively (Table 1).

Media fortified with PIC or 2,4-D, generally stimulated a more abundant callus formation (329.6–456.1 mg fresh weight, FW) than NAA (134.4–246.9 mg), while calli regenerated in the presence of IAA were of the lowest FW (9.0–25.7 mg, Table 1, Supplementary Figure S1B). In addition, leaf explants produced a higher mean FW of callus (326.5 mg), followed by petioles (261.6 mg), and internodes (150.8 mg). Maximal biomass of callus was found in the leaf explants cultured in M9 (0.5 mg·L^−1^ BA + 0.5 mg·L^−1^ 2,4-D) medium (642.8 mg). In contrast, calli produced on internodes had a higher share of dry weight (DW, 14.3%) compared to leaf-derived biomass (12.5%, Table 1). The presence of NAA, 2,4-D, or PIC had a negative impact on the DW in calli (Supplementary Figure S1B). The highest share of DW was found in internode explants cultured in the M4 (1.0 mg·L^−1^ BA + 2.0 mg·L^−1^ IAA) medium (30.8%). There was no impact of AXs and CKs ratio on the general callus regeneration frequency, callus FW, and DW (Table 2).

**Figure 1:**
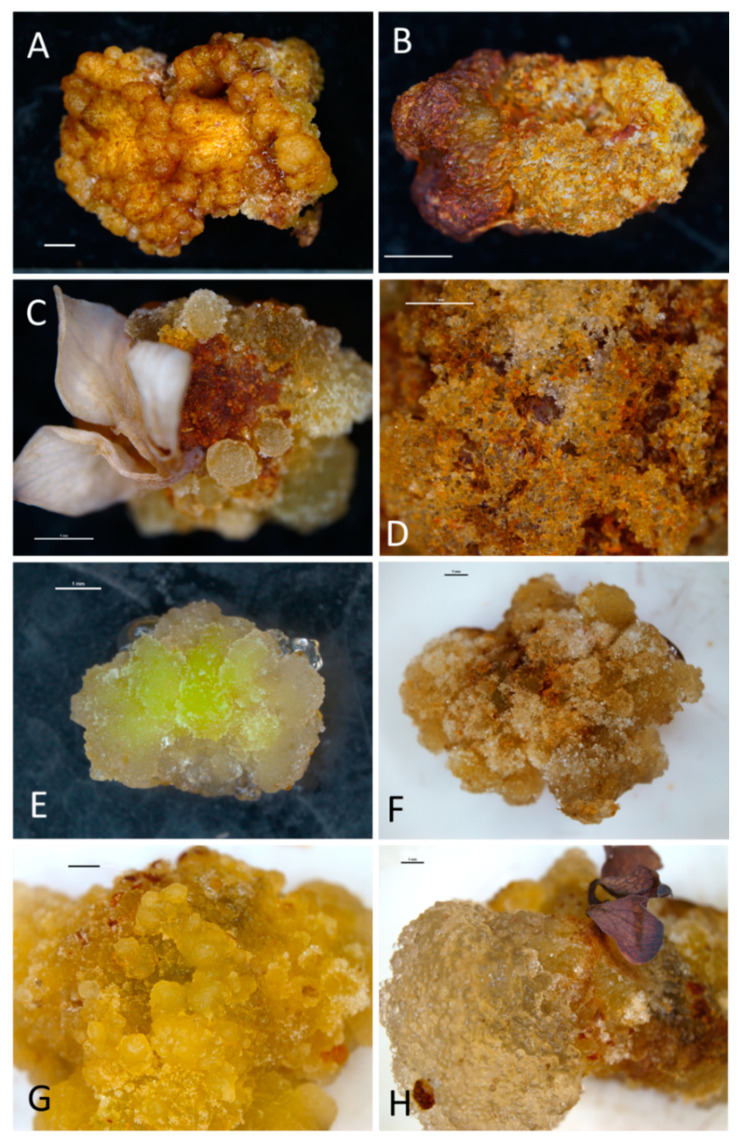
In vitro callogenesis in bleeding heart ‘Alba’ after 10 weeks of culture: (**A**)—embryogenic callus formed on a leaf petiole cultured in the MS medium with 0.5 mg·L^−1^ BA and 2.0 mg·L^−1^ IAA (M2). (**B**)—non-embryogenic callus on an internode in MS medium with 0.5 mg·L^−1^ BA and 1.0 mg·L^−1^ IAA (M1). (**C**)—embryogenic callus and its structure (**D**) on a whole-leaf explant in MS medium with 1.0 mg·L^−1^ BA and 1.0 mg·L^−1^ NAA (M8). (**E**)—non-embryogenic callus developed on an internode in MS medium with 0.5 mg·L^−1^ BA and 0.5 mg·L^−1^ NAA (M5). (**F**)—callogenesis on an internode in MS medium with 1.0 mg·L^−1^ BA and 0.5 mg·L^−1^ 2,4-D (M11). (**G**)—embryogenic callus on a petiole in MS medium with 0.5 mg·L^−1^ BA and 1.0 mg·L^−1^ PIC (M14). (**H**)—transparent non-embryogenic callus on a leaf in MS medium with 1.0 mg·L^−1^ BA and 1.0 mg·L^−1^ PIC (M16). Bar = 1 mm. 2,4-D, 2,4-dichlorphenoxyacetic acid; BA, 6-benzyladenine; IAA, indole-3-acetic acid; M, medium; MS, Murashige and Skoog (1962); NAA, 1-naphthaleneacetic acid; PIC, picloram.

The produced calli were usually firm and compact but differed in color. Callus produced on the IAA-supplemented medium was bronze-red (Figure 1A,B), in the presence of NAA—yellow-green-brown (Figure 1C–E), in the presence of 2,4-D—white-brown (Figure 1F), and in the PIC-supplemented medium—yellow-green (G,H).

**Figure 2:**
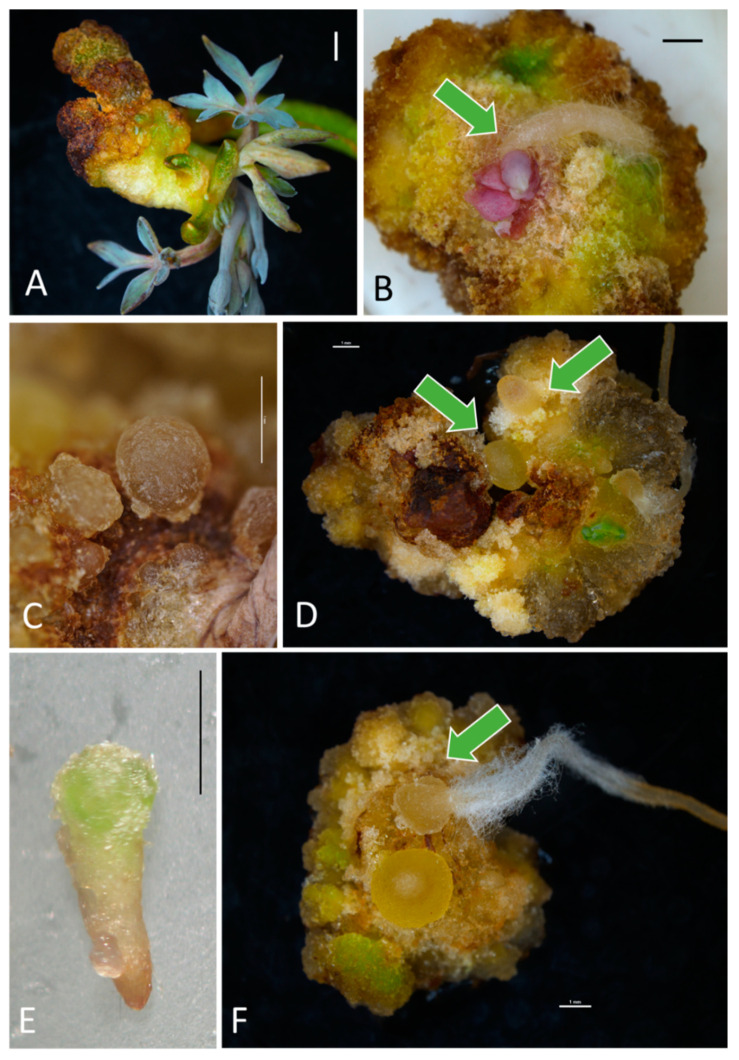
Adventitious organogenesis and embryogenesis in bleeding heart ‘Alba’ after 10 weeks of in vitro culture: (**A**)—adventitious shoots regenerating from an internode cultured in MS medium with 1.0 mg·L^−1^ BA and 1.0 mg·L^−1^ IAA (M3). (**B**)—adventitious root (arrow) regenerating on a leaf in MS medium with 0.5 mg·L^−1^ BA and 1.0 mg·L^−1^ NAA (M6). (**C**)—somatic embryo at a globular stage regenerating on leaf explant in MS medium with 0.5 mg·L^−1^ BA and 1.0 mg·L^−1^ 2,4-D (M10). (**D**)—somatic embryo at an early torpedo stage (arrows) on petiole in MS medium with 0.5 mg·L^−1^ BA and 1.0 mg·L^−1^ NAA (M6). (**E**)—somatic embryo at late torpedo/maturity developmental stage from a leaf in MS medium with 0.5 mg·L^−1^ BA and 1.0 mg·L^−1^ NAA (M6). (**F**)—germination of a somatic embryo (arrow) on petiole in MS medium with 0.5 mg·L^−1^ BA and 1.0 mg·L^−1^ NAA (M6). Bar = 1 mm. 2,4-D, 2,4-dichlorphenoxyacetic acid; BA, 6-benzyladenine; IAA, indole-3-acetic acid; M, medium; MS, Murashige and Skoog (1962); NAA, 1-naphthaleneacetic acid.

Among the 550 inoculated explants, only 14 (2.5%) regenerated indirectly a total of 36 adventitious shoots, usually in the presence of IAA (88.6% of all shoots) and at the cutting site (Figure 2A). Similarly, only 4.5% of explants formed indirectly adventitious roots (Figure 2B) mostly in NAA-supplemented media (72% of all roots. Data not shown). Explants cultured in the PGR-free control medium (M0) turned white or brown and died without any morphogenetic response.

### 2.2. Morphogenetic Response of Explants—Somatic Embryogenesis

Both factors tested (explant type and medium composition) affected the embryogenic potential of the bleeding heart (Table 3). Leaves formed embryogenic callus most often (54.3% of explants), which was followed by petioles (45.1%) and internodes (23.6%, Table 3). 

The regeneration of embryos was indirect. IAA and NAA were generally less effective in stimulating SE (0.0–40.7% efficiency, regardless of explant type) than 2,4-D or PIC (40.7–92.6%, Table 3, Supplementary Figure S1A). The dominance of AXs over CKs promoted SE (59.3% embryogenic calli and 4.8 embryos per explant), while CKs predominance declined the frequency of embryogenic callus formation (28.7%) and the number of regenerating embryos (2.2, Table 2). Maximal 100% SE efficiency was reported with leaf explants cultured in media M9 (0.5 mg·L^–1^ BA + 0.5 mg·L^–1^ 2,4-D) and M14 (0.5 mg·L^–1^ BA + 1.0 mg·L^–1^ PIC) as well as petioles in the M14 medium (Table 3). The latter experimental object provided the maximal number of somatic embryos per inoculated explant (11.4) even though several other combinations were also effective. Most of the somatic embryos were at the early globular stage (about 60%, Figure 2C) of development, even though more advanced torpedo (35%, Figure 2D,E) and maturity stage (5%, Figure 2F) could also be distinguished. Embryos were creamy-yellow, yellow-green or, less-often, brown (mostly in IAA-supplemented media). They had a bipolar structure (evident in more mature embryos). 

In contrast, internodes produced more non-embryogenic callus (mean of 60.4%) than other explant types (34.6–38.9%, data not shown). Also, IAA and NAA auxins favored non-embryogenic callus development (55.6–65.5%, Figure S1A).

### 2.3. Spectral Analysis of Primary and Secondary Metabolites in Callus

**Figure 3:**
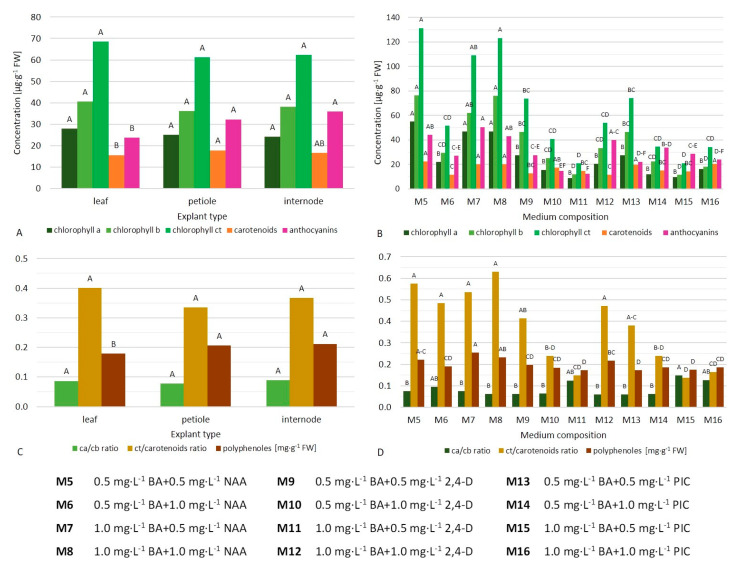
Main effects of explant type (**A**,**C**) and medium composition (**B**,**D**) (irrespectively) on the content of primary and secondary metabolites in callus of the bleeding heart ‘Alba’ after 10 weeks of in vitro culture.

**Figure 4:**
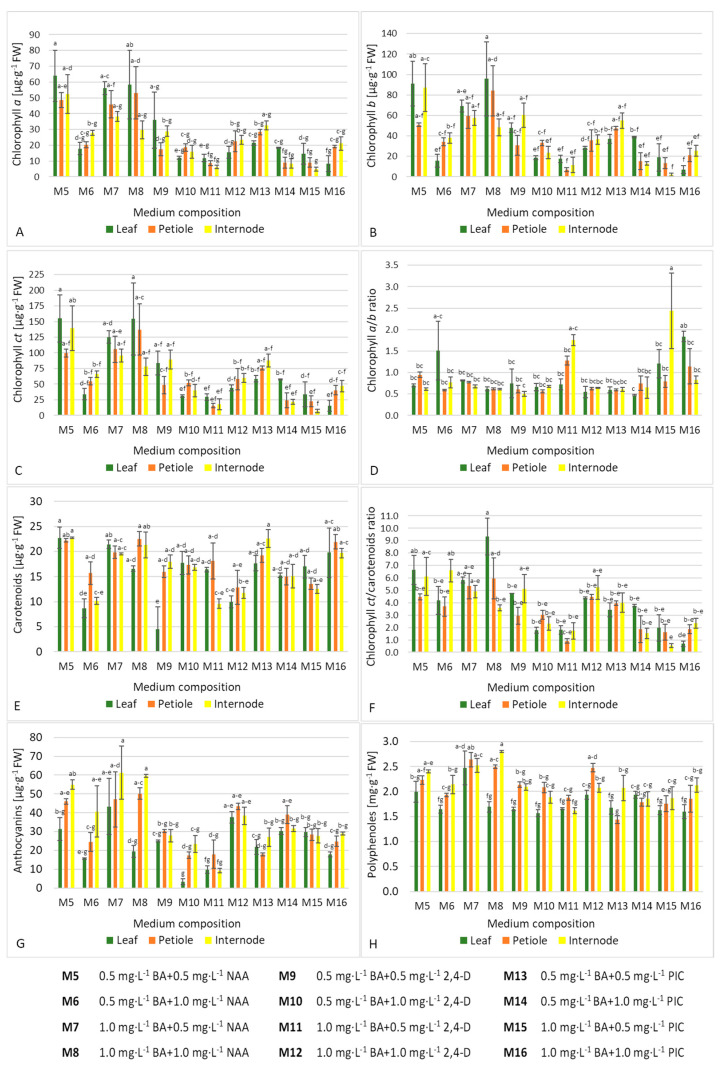
Effect of medium composition and explant type interaction on the content of primary (**A**–**D**) and secondary metabolites (**E**–**H**) in callus of bleeding heart ‘Alba’ after 10 weeks of in vitro culture.

Calli cultured on media with NAA usually contained a higher content of chlorophyll *a*, *b*, and *ct* (except for medium M6 with 0.5 mg·L^−1^ BA and 1.0 mg·L^−1^ NAA, Figure 3B, Supplementary Figure S1C). The highest content of chlorophyll *a* (58.44–63.95 µg·g^–1^ FW), chlorophyll *b* (91.09–95.72 µg·g^−1^ FW), and chlorophyll *ct* (154.16–155.04 µg·g^−1^ FW) were found in calli produced on leaf explants in media M5 (0.5 mg·L^−1^ BA + 0.5 mg·L^−1^ NAA) and M8 (1.0 mg·L^−1^ BA + 1.0 mg·L^−1^ NAA) (Figure 4A–C). In contrast, internodes cultured in M15 (1.0 mg·L^–1^ BA + 0.5 mg·L^–1^ PIC) medium produced the lowest chlorophyll content (6.91 µg *ct* per gram FW). A balanced ratio between CKs and AXs promoted chlorophyll biosynthesis (70.4–93.0 µg·g^−1^ FW, Table 4).

Only calli from media M11 (1.0 mg·L^−1^ BA + 0.5 mg·L^−1^ 2,4-D), M15 (1.0 mg·L^−1^ BA + 0.5 mg·L^−1^ PIC), and M16 (1.0 mg·L^−1^ BA + 1.0 mg·L^−1^ PIC) had a chlorophyll *a*/*b* ratio above 1.0 (Figure 3D). The highest value of this parameter was found in internode-derived calli in M15 (1.0 mg·L^−1^ BA + 0.5 mg·L^−1^ PIC) medium (2.44), while the lowest in callus produced on leaves in M14 (0.5 mg·L^−1^ BA + 1.0 mg·L^−1^ PIC) medium (0.47) and internodes in M9 (0.5 mg·L^−1^ BA + 0.5 mg·L^−1^ 2,4-D) medium (0.50) (Figure 4D). No impact of CKs and AXs ratio on this parameter was reported (Table 4).

Callus produced from leaf petioles contained more carotenoids (mean 17.84 µg·g^−1^ FW) than from whole-leaf explants (15.64 µg·g^−1^ FW, Figure 3A). In addition, the presence of NAA and PIC usually had a positive impact on this parameter, regardless of the explant type (Figure 3B, Supplementary S1C). The highest value of this parameter for whole-leaf-derived callus was observed in M5 (0.5 mg·L^−1^ BA + 0.5 mg·L^−1^ NAA) medium (22.71 µg·g^−1^ FW), for internode-derived in M5 and M13 (0.5 mg·L^−1^ BA + 0.5 mg·L^−1^ PIC) media (22.58–22.69 µg·g^−1^ FW), and for petiole-derived callus in medium M8 (1.0 mg·L^−1^ BA + 1.0 mg·L^−1^ NAA) (22.52 µg·g^−1^ FW, Figure 4E). Callus formed on leaves in the M9 (0.5 mg·L^−1^ BA + 0.5 mg·L^−1^ 2,4-D) medium was the least abundant in those pigments (4.5 µg·g^−1^ FW). Balanced low concentrations of cytokinin and auxin were preferable for the biosynthesis of carotenoids (Table 4).

Auxin NAA increased the value of total chlorophyll (*ct*) to carotenoids ratio (Figure 3D, Supplementary Figure S1D). The highest and lowest values of this coefficient were reported in leaf-derived callus on medium M8 (1.0 mg·L^−1^ BA + 1.0 mg·L^−1^ NAA) (9.34) and callus formed on internodes in medium M15 (1.0 mg·L^−1^ BA + 0.5 mg·L^−1^ PIC) (0.53), respectively (Figure 4F). Low concentrations of PGRs increased this parameter positively (Table 4).

Calli produced from internodes and petioles generally contained more anthocyanins (mean 32.25–35.86 µg·g^−1^ FW) than those from whole-leaf explants (23.77 µg·g^−1^ FW, Figure 3A). Similarly, the presence of NAA increased the concentration of those pigments, except for medium M6 (0.5 mg·L^−1^ BA + 1.0 mg·L^−1^ NAA) (Figure 3B, Supplementary Figure S1C). The highest content of anthocyanins was found in internode-derived calli in media M7 (1.0 mg·L^−1^ BA + 0.5 mg·L^−1^ NAA) and M8 (1.0 mg·L^−1^ BA + 1.0 mg·L^−1^ NAA) (59.54–61.26 µg·g^−1^ FW, Figure 4G). In contrast, leaf-derived callus in the M10 (0.5 mg·L^−1^ BA + 1.0 mg·L^−1^ 2,4-D) medium contained the lowest concentration of those pigments (3.40 µg·g^−1^ FW).

Petiole-derived and internode-derived calli had more polyphenols (mean 2.06–2.12 mg·g^−1^ FW) compared to those formed on whole leaves (1.79 mg·g^−1^ FW, Figure 3C). Media with NAA usually increased the production of those compounds (M5 with 0.5 mg·L^−1^ BA and 0.5 mg·L^−1^ NAA, M7 with 1.0 mg·L^−1^ BA and 0.5 mg·L^−1^ NAA, and M8 with 1.0 mg·L^−1^ BA and 1.0 mg·L^−1^ NAA; Figure 3D, Figure S1D). The content of polyphenols in callus from internodes cultured in medium M8 was nearly two-fold higher than in callus formed on petioles in medium M13 with 0.5 mg·L^−1^ BA and 0.5 mg·L^−1^ PIC (2.80 and 1.44 µg·g^−1^ FW, respectively, Figure 4H).

There was no impact of CKs and AXs ratio on the content of anthocyanins and polyphenols in the callus of the bleeding heart (Table 4).

Low (|*r*| ≤ 0.3) or moderate (0.3 < |*r*| ≤ 0.5) but always negative correlations between the content of metabolites tested and the share of explants forming embryogenic callus/number of somatic embryos per inoculated explant were observed (Table 5). On the other hand, there was a positive association between the frequency of non-embryogenic callus formation and the number of biochemical compounds. In addition, the production of various metabolites was positively correlated with each other (*r* = 0.20–0.93).

## 3. Discussion

Morphogenesis in vitro is a complex process affected by several endogenous and external factors with cumulative effects expressing the embryogenic and organogenic potential in explants that has not been fully explained to date [20]. It is widely used for reproduction and breeding purposes in numerous (floricultural) crops but adventitious organogenesis in the bleeding heart has not been elucidated. This is the first report on exploring morphogenetic events from non-meristematic explants in *L. spectabilis.*

### 3.1. Callogenesis, Adventitious Organogenesis, and Embryogenesis in the Bleeding Heart

Non-meristematic explants, such as whole leaves, leaf petioles, and internodes, were used in the present study since they are the easiest and most abundant to obtain. It was found that most of the indirectly regenerating adventitious shoots were produced on the cutting site of the explant. This could be a result of mechanical stimuli, which is known to promote the regenerability of the plant tissue. A similar phenomenon was observed by Tymoszuk et al. [21], with transversely-cut ligulate florets of chrysanthemum. Unfortunately, the efficiency of caulogenesis was surprisingly low with the bleeding heart (a total of 36 adventitious shoots were produced), even though media fortified with cytokinins and auxins were successfully utilized with numerous other plant genera [16,22]. For example, 7.9–17.4 adventitious shoots per explant were reported for *Mammillaria perbella* Hildm. ex K. Schum. and *M. orcutii* Boed. cultured in MS medium with 1.0 mg·L^−1^ IAA and 10.0 mg·L^−1^ KIN by Ramirez-Malagon et al. [23]. Those observations classify *L. spectabilis* as a species “difficult” to manipulate in vitro. The efficiency of adventitious shoots regeneration in *Meconopsis paniculata* D. Don. on media supplemented with different auxins (NAA, IAA, IBA, 2,4-D) and cytokinins (BA, KIN) ranged from 1 to 7 per explant [24]. Similarly, cells and tissues of other systematically related species representing Papaveraceae family, e.g., *Chelidonium majus* L., *Eschscholtzia californica* Cham., and *Papaver somniferum* L., have proven to be difficult to culture [25]. Problems with establishing efficient culture systems were reported with several woody species [26], but they are not so often reported with herbaceous plants [27]. The scarce number of adventitious shoots produced in a gross number of PGR combinations tested (including the separate application of BA, KIN, IAA, NAA, and PIC, unpublished data) could result from the small size of bleeding heart explants and indicate the need for evaluating other explant types, e.g., flower petals, roots, or a thin cell layer culture, which worked well with other ornamentals, such as chrysanthemum [28]. Another possibility is the utilization of less popular media types (e.g., Schenk and Hildebrandt [29] medium, SH) and/or PGRs such as gibberellins, jasmonates, brassinosteroids, polyamines, and strigolactones. The use of structural analogs of BA, e.g., 6-benzyl-adenine riboside (BAR) or (meta-)topolin (TOP), effective with *Sesamum indicum* L. [30], which might also enhance the in vitro regeneration frequency of a bleeding heart.

On the other hand, SE in *L. spectabilis* was much more effective. The produced embryos had a typical morphology as those described in several other plant species [9,16,28]. The highest mean number of somatic embryos (11.4 per inoculated explant in MS with 0.5 mg·L^−1^ BA and 1.0 mg·L^−1^ PIC) is quite high. As for chrysanthemum, a maximal of 5.7 embryos per explant (with 85% regeneration frequency) were produced in the MS medium with 4.0 mg·L^−1^ 2,4-D and 2.0 mg·L^−1^ KIN [21]. In yellow horned poppy (*Glaucium flavum* Crantz.), the efficiency of somatic embryogenesis reached 17.2 embryos per explants in the MS medium supplemented with 1.0 mg·L^−1^ 2,4-D, 0.5 mg·L^−1^ TDZ and 0.2 mg·L^−1^ BA [31]. Somatic embryogenesis is a powerful tool since it allows the production of complete functional embryos, without fertilization, potentially from each somatic cell of the explant under appropriate conditions. Conversion of the embryos into complete plantlets seems a bottleneck in *L. spectabilis*, but an additional subculture on an embryo-germination medium is often a necessity [32]. Perhaps with bleeding heart ‘Alba’, a subculture of somatic embryos to a KIN-supplemented medium would be effective for embryo maturation, as reported in other studies [8]. 

Auxins and cytokinins are considered the principal PGRs involved in modulating the occurrence of signaling events during morphogenesis. The results obtained in this case allowed us to decipher that BA had a pleiotropic effect in the bleeding heart, as it caused different morphogenetic responses depending on its concentration and presence of other PGRs, including callus formation, indirect shoot organogenesis, and/or SE. On the contrary, IAA showed an inhibiting role in callus and somatic embryo formation, which stimulates adventitious shoot regeneration. Similar results were reported by García-Pérez et al. [12] with the *Bryophyllum* subgenus. Auxin NAA enhanced root formation (data not shown), which coincides with the previous findings with a bleeding heart [9]. As for SE, 2,4-D and PIC were superior to obtain a high regeneration frequency of embryogenic callus and somatic embryos. Synthetic auxins are very effective during the initiation and proliferation of embryogenic cultures (by initiating cell division activity in the procambial cells of explant) compared to natural auxins, but block the expression of genes involved in the transition to the more mature stages [32].

The balance between AXs and CKs determines the morphogenetic response in explants. Normally, the dominance of CKs over AXs promotes cell division and shoot elongation. A balanced ratio between CKs and AXs favors callus formation and embryogenesis, and AXs predominance results in root formation and elongation [33]. In the present study, there was no impact of PGRs ratio on the general callus regeneration frequency and its weight, although AXs predominance stimulated SE, which coincides with the findings of other authors, who used increased concentrations of AXs as promoters of cell redifferentiation [32].

It is well known that somatic embryos develop mostly from a single totipotent somatic cell in the explant (the pre-embryogenic determined cells in the direct path) or in pro-embryogenic masses re-differentiated from the parenchyma-like callus cells in the indirect path [32]. Nonetheless, various explant types have different embryogenic potential, depending on the species. In the present study, it was found that whole-leaf explants are superior for the establishment of callogenesis and SE in the bleeding heart, whereas internodes are the least efficient. Callus derived from leaf explants is permeated by vascular tissue, which favors embryo development and explains the present findings [32]. Leaf explants were also successfully utilized in achieving direct somatic embryogenesis in *Oncidium Gower* Ramsey and subsequent plant regeneration [34]. In contrast, nodal segments were more efficient than whole leaves, half leaves, petioles, and root segments in *Anthurium andreanum* Linden [35]. The high-frequency embryogenesis of leaf cells in the bleeding heart is strong evidence of their totipotency, and further modification of the protocol for plant formation could be useful for the mass reproduction and transformation of selected elite lines.

### 3.2. Biosynthesis of Metabolites in Callus

Plants are a tremendous source for the discovery of new products with medicinal and industrial importance. Approximately 25% of all drugs approved by the Food and Drug Administration proceed from plant sources [36]. For example, in *Artemisia annua* L., tissue cultures are used for the synthesis of artemisinin [37]. The present study, for the first time, evaluated the impact of various exogenous factors on the synthesis of different metabolites in callus cultures of the bleeding heart. 

Chlorophylls and carotenoids are involved in photosynthesis, but their derivatives consistent with cancer prevention include antioxidant and antimutagenic activity. They are also used as a food coloring agent [38,39]. Anthocyanins are secondary metabolites determining the pink, purple, and blue color of plant organs, valuable in medicine due to anticancer, antioxidant, and other health-promoting properties [40]. There is also substantial evidence that specific polyphenols, normally involved in the plant defense system, benefit human health status, especially for the prevention and management of certain chronic diseases, including obesity, type 2 diabetes, and neurodegenerative diseases [41]. Therefore, establishing an effective tissue culture system of their production is justified and needed.

The present study showed that metabolite biosynthesis in *L. spectabilis* can be controlled by medium variables and explant type, which is in agreement with the reports of other authors [18]. Secondary metabolites are known to play a major role in the adaptation of plants to their environment [18], which could explain the observed variation in their content.

When an explant is inoculated in vitro, diverse responses are expected, depending on its origin. According to Tarrahi and Rezanejad [42], the highest anthocyanin and chlorophyll yield in *Rosa* spp. were obtained in vegetative calluses, especially in the leaf and stem, compared with flower calluses. In the present study, calli derived from petioles and internodes contained more anthocyanins, carotenoids, and polyphenols than those produced on whole leaves, but did not differ in terms of chlorophyll content. This could be the effect of mechanical injury during explant excision, which stimulated the production of stress-related metabolites [43].

Cytokinins play an important role in the development and structural differentiation of chloroplasts [15]. Consequently, increased BA concentration raised the content of chlorophyll and carotenoids in the leaves of *Gerbera jamesonii* Bolus cultured in vitro [44]. Callus cultures of the bleeding heart, however, seem to respond differently since the highest levels of chlorophylls and carotenoids were found in the experimental combination with the lowest concentrations of PGRs. There was also no impact of various PGRs ratios on the chlorophyll *a/b* ratio or anthocyanins and polyphenols content. In contrast, NAA elevated the levels of all metabolites analyzed in this study, which suggests strong antioxidant activities in calli. Similarly, NAA proved to stimulate isoflavone production in *Genista tinctoria* L. calli at 5.0 mg·L^−1^ when applied jointly with 0.5 mg·L^−1^ cytokinin [45]. Perhaps using a light factor instead of PGRs combination would allow for even more effective control of pigment synthesis in the bleeding heart, as reported by Cioć et al. in gerbera [44].

In the present study, a negative correlation between the content of chemical compounds in callus and SE efficiency was found (Table 5). To the best of our knowledge, this is the first study to analyze such a relationship. Due to the consistent results, it seems that measurement of callus chemical compositions can be used as a marker for evaluating the occurrence of somatic embryogenesis in the bleeding heart as well as in other plant species. In contrast, acquisition of metabolite synthesis should be performed with the use of non-embryogenic callus. Since a positive correlation between the concentrations of all analyzed compounds was observed (*r* = 0.20–0.93), the in vitro production of those metabolites can be performed simultaneously.

Further studies should focus on the use of exogenous melatonine [46,47], elicitors (exogenous IBA, fungal elicitors, and oligochitosan), and/or precursor feeding in the overproduction of valuable metabolites in the bleeding heart [48]. 

## 4. Materials and Methods

### 4.1. Plant Material, Media Preparation, and General Culture Conditions

The Murashige and Skoog [49] medium (MS) was used in the experiment, modified by increasing half the concentration of calcium II chloride (CaCl_2_·6H_2_O), iron sulfate (FeSO_4_), and Na_2_EDTA·2H_2_O, supplemented with 3% (*w/v*) sucrose, and solidified with 0.8% (*w/v*) agar (Biocorp, Warsaw, Poland). A cytokinin (BA) and auxin (IAA, NAA, 2,4-D, or PIC) were added into the culture medium in combination, at the concentration of 0.5; 1.0, or 2.0 mg·L^−1^ each. Based on the qualitative and quantitative composition of PGRs, the media were assigned the following symbols: M0 (a PGR-free control medium), M1 (0.5 mg·L^−1^ BA + 1.0 mg·L^−1^ IAA), M2 (0.5 mg·L^−1^ BA + 2.0 mg·L^−1^ IAA), M3 (1.0 mg·L^−1^ BA + 1.0 mg·L^−1^ IAA), M4 (1.0 mg·L^−1^ BA + 2.0 mg·L^−1^ IAA), M5 (0.5 mg·L^−1^ BA + 0.5 mg·L^−1^ NAA), M6 (0.5 mg·L^−1^ BA + 1.0 mg·L^−1^ NAA), M7 (1.0 mg·L^−1^ BA + 0.5 mg·L^−1^ NAA), M8 (1.0 mg·L^−1^ BA + 1.0 mg·L^−1^ NAA), M9 (0.5 mg·L^−1^ BA + 0.5 mg·L^−1^ 2,4-D), M10 (0.5 mg·L^−1^ BA + 1.0 mg·L^−1^ 2,4-D), M11 (1.0 mg·L^−1^ BA + 0.5 mg·L^−1^ 2,4-D), M12 (1.0 mg·L^−1^ BA + 1.0 mg·L^−1^ 2,4-D), M13 (0.5 mg·L^−1^ BA + 0.5 mg·L^−1^ PIC), M14 (0.5 mg·L^−1^ BA + 1.0 mg·L^−1^ PIC), M15 (1.0 mg·L^−1^ BA + 0.5 mg·L^−1^ PIC), and M16 (1.0 mg·L^−1^ BA + 1.0 mg·L^−1^ PIC) (Table 1). All PGRs were provided by Sigma-Aldrich^®^, St. Louis, MO, USA. The pH was adjusted to 5.8 after adding all media components (Chemia, Bydgoszcz, Poland), prior to sterilization at 105 kPa and 121 °C for 20 min. The medium (40 mL) was poured into 350-mL glass jars and sealed with plastic caps.

The 10-week-old in vitro-derived shoots of *Lamprocapnos spectabilis* (L.) Fukuhara ‘Alba’ were used as the donor plant material. Whole leaves (7–10 mm-long), leaf petioles (3–5 mm), and internodes (1–2 mm) were excised from the central part of shoots and inoculated polarly (leaves) or vertically (petioles and internodes) in the modified MS medium with nine explants per jar. Each jar was considered a single repetition. The experiment was repeated thrice.

The cultures were kept in the growth chamber at 24 ± 1 °C, under 16-h photoperiod conditions and photosynthetic photon flux density of approximately 30 µmol·m^−2^·s^−1^ provided by standard cool daylight TLD 54/36W fluorescent tubes with a color temperature of 6200 K (Koninklijke Philips Electronics N.V., Eindhoven, the Netherlands).

### 4.2. Evaluation of a Morphogenetic Response in Explants

After 10 weeks of culturing, the share of explants forming callus, adventitious shoots, and roots was counted. The fresh weight and share of dry weight of calli (dried in a laboratory oven at 105 °C for 3 h) were also evaluated. The share of inoculated explants forming somatic embryos, and the number of embryos per one inoculated explant was included.

### 4.3. Spectral Assay

The spectral analysis of metabolites was performed for in vitro regenerated calli in the 10th week of the culture in three repetitions. Twelve experimental combinations (M5–M16) were included in the array. Due to an insufficient amount of callus produced in combinations M0–M5 (PGR-free control and media with BA and IAA), those experimental objects were not included in the study.

Chlorophylls and carotenoids were extracted from fresh calli, as described by Lichtenthaler [50] using 100% acetone and 50 mg of tissue samples. Anthocyanins were extracted using 200 mg callus samples and methanol containing 1% HCl (*v/v*), according to the Harborne [51] method. The same extract was used to analyze the total phenolic content, according to the Folin-Ciocalteau procedure [52]. The total phenolic content was calculated using gallic acid as the calibration standard. 

The spectrophotometric analysis of extracts was performed in a spectrophotometer SmartSpec PlusTM (BioRad, Hercules, CA, USA) at specific wavelengths (λ_max_): for carotenoids at 470 nm, for anthocyanins at 530 nm, for chlorophyll *a* and *b* at 645 and 662 nm, and for phenolics at 765 nm, respectively. The content of pigments and phenolics was calculated per gram of fresh matter.

### 4.4. Statistical Analysis

The experiment was set up in a completely randomized design. To obtain the normal distribution of the data expressed as a percentage, the Freeman-Tukey transformation was used. Data were statistically verified by applying Statistica 12.0 (StatSoft, Warsaw, Poland) software. The analysis of variance (ANOVA) was performed and means were evaluated with the Newman-Keuls test at the significance level of *p* ≤ 0.05. Tables with results provide real, untransformed numerical data (mean ± standard error) with the alphabet indicating the homogeneous groups. 

Associations between the content of chemical compounds in callus and the share of explants forming (non-)embryogenic callus and number of somatic embryos per explant were also analyzed, based on the Pearson correlation coefficient (*r*, *p* ≤ 0.05).

## 5. Conclusions

This is the maiden research on the in vitro culture systems adjusted from non-meristematic explants in *Lamprocapnos spectabilis*. Due to the multifactorial behavior, the design of a universal protocol for plant regeneration is a challenging task. The induction of adventitious organogenesis seems difficult and not efficient in the bleeding heart. Somatic embryogenesis is more promising, especially when using whole-leaf or petiole explants and 2,4-D or PIC-fortified media. Medium composition affects not only the production of biomass but also the biosynthesis of metabolites. Calli cultured on media with NAA usually contain a higher content of primary and secondary metabolites, but produce less somatic embryos. There is also a significant impact of the explant type on the content of anthocyanins, polyphenols, and carotenoids in callus. Further studies should focus on the application of elicitor stimulation in the overproduction of economically important compounds.

## Figures and Tables

**Table 1:** Effect of medium composition and explant type on the share of bleeding-heart explants forming a callus, a callus fresh weight, and a share of dry weight in the callus.

Medium Symbol	Plant Growth Regulator (mg·L^−1^)			Explant Type			Mean
	BA	Auxin		Leaf	Petiole	Internode	
				**Callus (%)**			
M1	0.5	IAA	1.0	66.7 ab	66.7 ab	44.4 b	59.3 C
M2	0.5		2.0	88.9 ab	88.9 ab	77.8 ab	85.2 A–C
M3	1.0		1.0	55.5 b	77.8 ab	77.7 ab	70.4 A–C
M4	1.0		2.0	55.5 b	77.8 ab	66.7 ab	66.7 BC
M5	0.5	NAA	0.5	88.9 ab	55.6 ab	77.8 ab	74.1 A–C
M6	0.5		1.0	88.9 ab	77.8 ab	77.8 ab	81.5 A–C
M7	1.0		0.5	100 a	77.8 ab	88.9 ab	88.9 AB
M8	1.0		1.0	100 a	77.8 ab	100 a	92.6 AB
M9	0.5	2,4-D	0.5	100 a	66.7 ab	77.8 ab	81.5 A–C
M10	0.5		1.0	100 a	100 a	88.9 ab	96.3 A
M11	1.0		0.5	77.8 ab	88.9 ab	88.9 ab	85.2 A–C
M12	1.0		1.0	100 a	100 a	100 a	100 A
M13	0.5	PIC	0.5	100 a	100 a	88.9 ab	96.3 A
M14	0.5		1.0	100 a	100 a	100 a	100 A
M15	1.0		0.5	100 a	88.9 ab	100 a	96.3 A
M16	1.0		1.0	100 a	100 a	88.9 ab	96.3 A
**Mean**				65.3 A	62.4 A	62.1 A	
				**Callus fresh weight [mg]**			
M1	0.5	IAA	1.0	6.0 ± 2.2 g	8.3 ± 4.4 g	12.9 ± 6.3 g	9.0 E
M2	0.5		2.0	12.6 ± 1.0 g	16.0 ± 5.8 g	34.0 ± 13.5 fg	20.9 E
M3	1.0		1.0	20.1 ± 7.2 g	19.6 ± 14.0 g	37.5 ± 30 fg	25.7 E
M4	1.0		2.0	41.8 ± 10.7 fg	12.9 ± 2.6 g	20.3 ± 4.7 g	25.0 E
M5	0.5	NAA	0.5	339.8 ± 70.8 a–g	147.2 ± 47.3 d–g	70.0 ± 10.3 e–g	185.6 CD
M6	0.5		1.0	313.6 ± 153.7 a–g	195.8 ± 50.5 c–g	231.4 ± 77.1 c–g	246.9 BC
M7	1.0		0.5	207.4 ± 81.0 c–g	140.4 ± 35.3 d–g	55.4 ± 25.1 e–g	134.4 C–E
M8	1.0		1.0	239.4 ± 74.9 c–g	99.3 ± 15.4 e–g	77.8 ± 10.2 e–g	138.8 C–E
M9	0.5	2,4-D	0.5	642.8 ± 157.0 a	466.9 ± 42.4 a–e	226.1 ± 15.9 c–g	442.6 A
M10	0.5		1.0	548.0 ± 86.4 a–c	376.1 ± 71.9 a–g	192.2 ± 55.2 c–g	372.1 AB
M11	1.0		0.5	617.3 ± 158.8 ab	521.7 ± 157.2 a–c	229.4 ± 31.8 c–g	456.1 A
M12	1.0		1.0	561.6 ± 90.5 a–c	199.2 ± 57.9 c–g	228.0 ± 28.7 c–g	329.6 AB
M13	0.5	PIC	0.5	372.7 ± 20.4 a–g	479.9 ± 27.2 a–d	208.7 ± 34.9 c–g	353.8 AB
M14	0.5		1.0	403.7 ± 31.4 a–f	552.5 ± 31.7 a–c	250.1 ± 16.9 b–g	402.1 AB
M15	1.0		0.5	340.8 ± 26.4 a–g	481.1 ± 48.7 a–d	280.0 ± 49.5 a–g	367.3 AB
M16	1.0		1.0	556.8 ± 134.4 a–c	537.0 ± 8.4 a–c	258.9 ± 85.1 b–g	450.9 A
**Mean**				326.5 A	261.6 B	150.8 C	
				**Callus dry weight [%]**			
M1	0.5	IAA	1.0	30.6 a	20.5 b–e	17.5 c–f	22.8 A
M2	0.5		2.0	22.4 b–d	24.5 a–c	24.0 a–c	23.6 A
M3	1.0		1.0	27.1 ab	25.5 ab	24.7 a–c	25.8 A
M4	1.0		2.0	16.1 d–g	27.3 ab	30.8 a	24.7 A
M5	0.5	NAA	0.5	10.0 f–i	14.4 e–i	10.3 f–i	11.6 BC
M6	0.5		1.0	9.5 f–i	10.6 f–i	14.0 e–i	11.4 BC
M7	1.0		0.5	11.9 f–i	14.1 e–i	9.2 f–i	11.7 BC
M8	1.0		1.0	11.7 f–i	13.7 e–i	13.8 e–i	13.0 B
M9	0.5	2,4-D	0.5	9.3 f–i	10.9 f–i	12.1 f–i	10.8 BC
M10	0.5		1.0	6.8 g–i	8.9 f–i	15.0 e–h	10.2 BC
M11	1.0		0.5	4.9 i	8.8 f–i	9.7 f–i	7.8 C
M12	1.0		1.0	10.1 f–i	6.1 hi	9.1 f–i	8.4 BC
M13	0.5	PIC	0.5	7.6 g–i	8.0 f–i	10.1 f–i	8.6 BC
M14	0.5		1.0	7.3 g–i	7.6 g–i	8.5 f–i	7.8 C
M15	1.0		0.5	7.8 f–i	9.2 f–i	11.1 f–i	9.4 BC
M16	1.0		1.0	6.9 g–i	7.5 g–i	8.1 f–i	7.5 C
**Mean**				12.5 B	13.6 AB	14.3 A	

Means ± standard errors in rows and columns followed by the same letter do not differ significantly according to the Newman-Keuls test at *p* ≤ 0.05. Upper-case letters refer to the main effects (irrespectively) while lower-case letters refer to the interaction between the two studied, independent variables. 2,4-D, 2,4-dichlorphenoxyacetic acid; BA, 6-benzyladenine; IAA, indole-3-acetic acid; M, medium; NAA, 1-naphthaleneacetic acid; PIC, picloram.

**Table 2:** Effect of balance between cytokinin and auxin (irrespective of type) on the morphogenetic response in *L. spectabilis*.

PGRs Concentration		Total Callus (%)	Callus FW (mg)	Callus DW (%)	Non-Embryogenic Callus (%)	Embryogenic Callus (%)	No. of Embryos Per Explant
**Cytokinin**	**Auxin**						
low	low	77.8 a	242.2 ± 37.3 a	13.4 a	41.7 ab	36.1 b	3.0 ± 0.7 ab
low	high	90.7 a	260.5 ± 34.1 a	13.3 a	31.5 b	59.3 a	4.8 ± 0.8 a
high	low	85.2 a	245.9 ± 38.0 a	13.7 a	56.5 a	28.7 b	2.2 ± 0.7 b
high	high	88.9 a	236.1 ± 36.7 a	13.4 a	48.8 ab	40.0 b	2.5 ± 0.5 ab

Means ± standard errors in columns followed by the same letter do not differ significantly according to the Newman-Keuls test at *p* ≤ 0.05. Low concentration of cytokinin/auxin: 0.5 mg·L^−1^ BA, NAA, 2,4-D or PIC, 1.0 mg·L^−1^ IAA. High concentration of cytokinin/auxin: 1.0 mg·L^−1^ BA, NAA, 2,4-D or PIC, 2.0 mg·L^−1^ IAA. 2,4-D, 2,4-dichlorphenoxyacetic acid; BA, 6-benzyladenine; DW, dry weight; FW, fresh weight; IAA, indole-3-acetic acid; NAA, 1-naphthaleneacetic acid; PGR, plant growth regulator; PIC, picloram.

**Table 3:** Effect of medium composition and explant type on the share of bleeding-heart explants forming embryogenic callus and a mean number of somatic embryos per inoculated explant.

Medium Symbol	Plant Growth Regulator (mg·L^−1^)			Explant Type			Mean
	BA	Auxin		Leaf	Petiole	Internode	
				**Embryogenic Callus (%)**			
M1	0.5	IAA	1.0	11.1 bc	22.2 a–c	0.0 c	11.1 DE
M2	0.5		2.0	44.4 a–c	33.3 a–c	0.0 c	25.9 CD
M3	1.0		1.0	22.2 a–c	0.0 c	0.0 c	7.4 DE
M4	1.0		2.0	11.1 bc	33.3 a–c	0.0 c	14.8 DE
M5	0.5	NAA	0.5	0.0 c	0.0 c	0.0 c	0.0 E
M6	0.5		1.0	55.6 a–c	44.5 a–c	22.2 a–c	40.7 B–D
M7	1.0		0.5	11.1 bc	0.0 c	0.0 c	3.7 DE
M8	1.0		1.0	69.5 a–c	22.2 a–c	0.0 c	30.6 CD
M9	0.5	2,4-D	0.5	100 a	55.6 a–c	33.3 a–c	63.0 A–C
M10	0.5		1.0	88.9 ab	88.9 ab	55.6 a–c	77.8 AB
M11	1.0		0.5	55.6 a–c	55.6 a–c	11.1 bc	40.7 B–D
M12	1.0		1.0	88.9 ab	22.2 a–c	11.1 bc	40.7 B–D
M13	0.5	PIC	0.5	77.8 a–c	66.7 a–c	66.7 a–c	70.4 AB
M14	0.5		1.0	100 a	100 a	77.8 a–c	92.6 A
M15	1.0		0.5	55.5 a–c	88.9 ab	44.4 a–c	63.0 A–C
M16	1.0		1.0	77.8 a–c	88.9 ab	55.6 a–c	74.1 AB
**Mean**				54.3 A	45.1 B	23.6 C	
				**No. of embryos per explant**			
M1	0.5	IAA	1.0	0.3 ± 0.3 cd	0.8 ± 0.4 b–d	0.0 d	0.4 E
M2	0.5		2.0	1.6 ± 0.3 b–d	1.1 ± 0.7 b–d	0.0 d	0.9 E
M3	1.0		1.0	1.8 ± 1.0 b–d	0.0 d	0.0 d	0.6 E
M4	1.0		2.0	0.4 ± 0.4 cd	0.4 ± 0.1 cd	0.0 d	0.3 E
M5	0.5	NAA	0.5	0.0 d	0.0 d	0.0 d	0.0 E
M6	0.5		1.0	2.0 ± 0.5 b–d	1.9 ± 1.0 b–d	0.3 ± 0.2 cd	1.4 DE
M7	1.0		0.5	0.1 ± 0.1 d	0.0 d	0.0 d	0.0 E
M8	1.0		1.0	2.2 ± 0.2 b–d	0.3 ± 0.3 cd	0.0 d	0.9 E
M9	0.5	2,4-D	0.5	9.2 ± 2.7 a–d	7.1 ± 3.6 a–d	2.3 ± 0.2 b–d	6.2 A–C
M10	0.5		1.0	8.9 ± 1.3 a–d	9.1 ± 3.1 a–d	3.9 ± 1.8 a–d	7.3 AB
M11	1.0		0.5	5.1 ± 4.2 a–d	4.3 ± 3.4 a–d	0.1 ± 0.1 d	3.2 C–E
M12	1.0		1.0	5.6 ± 0.3 a–d	2.1 ± 2.1 b–d	1.3 ± 1.3 b–d	3.0 C–E
M13	0.5	PIC	0.5	8.8 ± 3.5 a–d	4.7 ± 1.9 a–d	2.8 ± 0.9 b–d	5.4 BC
M14	0.5		1.0	9.7 ± 1.3 ab	11.4 ± 0.5 a	7.2 ± 2.1 a–d	9.4 A
M15	1.0		0.5	2.9 ± 2.2 b–d	9.3 ± 3.9 a–c	2.2 ± 0.6 b–d	4.8 B–D
M16	1.0		1.0	5.9 ± 1.1 a–d	6.3 ± 1.7 a–d	5.9 ± 3.4 a–d	6.0 A–C
**Mean**				4.0 A	3.7 A	1.6 B	

Means ± standard errors in rows and columns followed by the same letter do not differ significantly according to the Newman-Keuls test at *p* ≤ 0.05. Upper-case letters refer to the main effects (irrespectively). Lower-case letters refer to the interaction between the two studied independent variables. 2,4-D, 2,4-dichlorphenoxyacetic acid; BA, 6-benzyladenine; IAA, indole-3-acetic acid; M, medium; NAA, 1-naphthaleneacetic acid; PIC, picloram.

**Table 4:** Effect of balance between cytokinin and auxin (irrespective of type) on the biochemical activity in callus of *L. spectabilis*.

PGRs Concentration		Chlorophyll *ct* (µg·g^−1^ FW)	Chlorophyll a/b (µg·g^−1^ FW)	Carotenoids (µg·g^−1^ FW)	Chlorophyll *ct*/Carotenoids	Anthocyanins (µg·g^−1^ FW)	Polyphenols (mg·g^−1^ FW)
Cytokinin	Auxin						
low	low	93.0 ± 8.5 a	0.7 ± 0.04 a	18.4 ± 1.2 a	4.6 ± 0.4 a	31.3 ± 2.4 a	2.0 ± 0.07 a
low	high	42.2 ± 3.5 b	0.7 ± 0.09 a	14.6 ± 0.7 b	3.2 ± 0.4 ab	25.1 ± 2.7 a	1.9 ± 0.05 a
high	low	50.3 ± 9.0 b	1.2 ± 0.18 a	16.4 ± 0.8 ab	2.7 ± 0.4 b	30.5 ± 4.1 a	2.0 ± 0.09 a
high	high	70.4 ± 11.0 ab	0.8 ± 0.14 a	17.4 ± 1.1 ab	4.2 ± 0.6 ab	35.6 ± 2.7 a	2.1 ± 0.08 a

Means ± standard errors in columns followed by the same letter do not differ significantly, according to the Newman-Keuls test at *p* ≤ 0.05. Low concentration of cytokinin/auxin: 0.5 mg·L^−1^ BA, NAA, 2,4-D or PIC, 1.0 mg·L^−1^ IAA. High concentration of cytokinin/auxin: 1.0 mg·L^−1^ BA, NAA, 2,4-D or PIC, 2.0 mg·L^−1^ IAA. 2,4-D, 2,4-dichlorphenoxyacetic acid; BA, 6-benzyladenine; IAA, indole-3-acetic acid; NAA, 1-naphthaleneacetic acid; PGR, plant growth regulator; PIC, picloram.

**Table 5:** Magnitude of association between the content of chemical compounds in callus and the share of explants forming (non-)embryogenic callus, and the number of somatic embryos per explant, based on the Pearson correlation coefficient (*p* ≤ 0.05), regardless of medium composition and explant type.

Trait	1	2	3	4	5	6	7	8
1. Non-embryogenic callus	1.00							
2. Embryogenic callus	−0.87	1.00						
3. No. of embryos	−0.70	0.83	1.00					
4. Chlorophyll *a*	0.36	−0.39	−0.34	1.00				
5. Chlorophyll *b*	0.30	−0.33	−0.31	0.93	1.00			
6. Carotenoids	0.23	−0.27	−0.23	0.39	0.36	1.00		
7. Anthocyanins	0.39	−0.43	−0.32	0.33	0.32	0.20	1.00	
8. Polyphenols	0.41	−0.48	−0.36	0.33	0.33	0.28	0.78	1.00

Shade refers to the type of correlation: green colors represent positive correlation, orange colors represent negative correlation; color gradient refers to the strength of correlation (low: |r| ≤ 0.3, moderate: 0.3 < |r| ≤ 0.5, strong: 0.5 < |r| ≤ 0.7 or very strong: |r| > 0.7). Axes separate morphogenetic and biochemical traits.

## Supplementary Materials

Supplementary materials can be found at https://www.mdpi.com/1422-0067/21/16/5826/s1. Figure S1. Influence of auxin type on the morphogenetic (**A**,**B**) and biochemical response (**C**,**D**) of the bleeding heart explants, irrespective of cytokinin concentration and explant type.

## Abbreviations

2,4-D	2,4-dichlorphenoxyacetic acid
ANOVA	Analysis of variance
AXs	Auxins
BA	6-benzyladenine
BAR	6-benzyl-adenine riboside
ca	Chlorophyll *a*
cb	Chlorophyll *b*
CKs	Cytokinins
ct	Total chlorophyll
DW	Dry weight
FW	Fresh weight
IAA	Indole-3-acetic acid
IBA	Indole-3-butyric acid
KIN	Kinetin
M	Medium
MS	Murashige and Skoog (1962) medium
NAA	1-naphthaleneacetic acid
PGR	Plant growth regulator
PIC	Picloram
SE	Somatic embryogenesis
SH	Schenk and Hildebrandt (1972) medium
TDZ	Thidiazuron
TOP	Topolin
ZEA	Zeatin
