# Supplementary material for: Induction of Callogenesis, Organogenesis, and Embryogenesis in Non-Meristematic Explants of Bleeding Heart and Evaluation of Chemical Diversity of Key Metabolites from Callus
Source: Int J Mol Sci. 2020 Aug 13;21(16):5826. doi: 10.3390/ijms21165826 (PMC7461564; doi:10.3390/ijms21165826)
Supplement: Supplementary file 1 [file ijms-21-05826-s001.pdf]

Supplementary Materials:

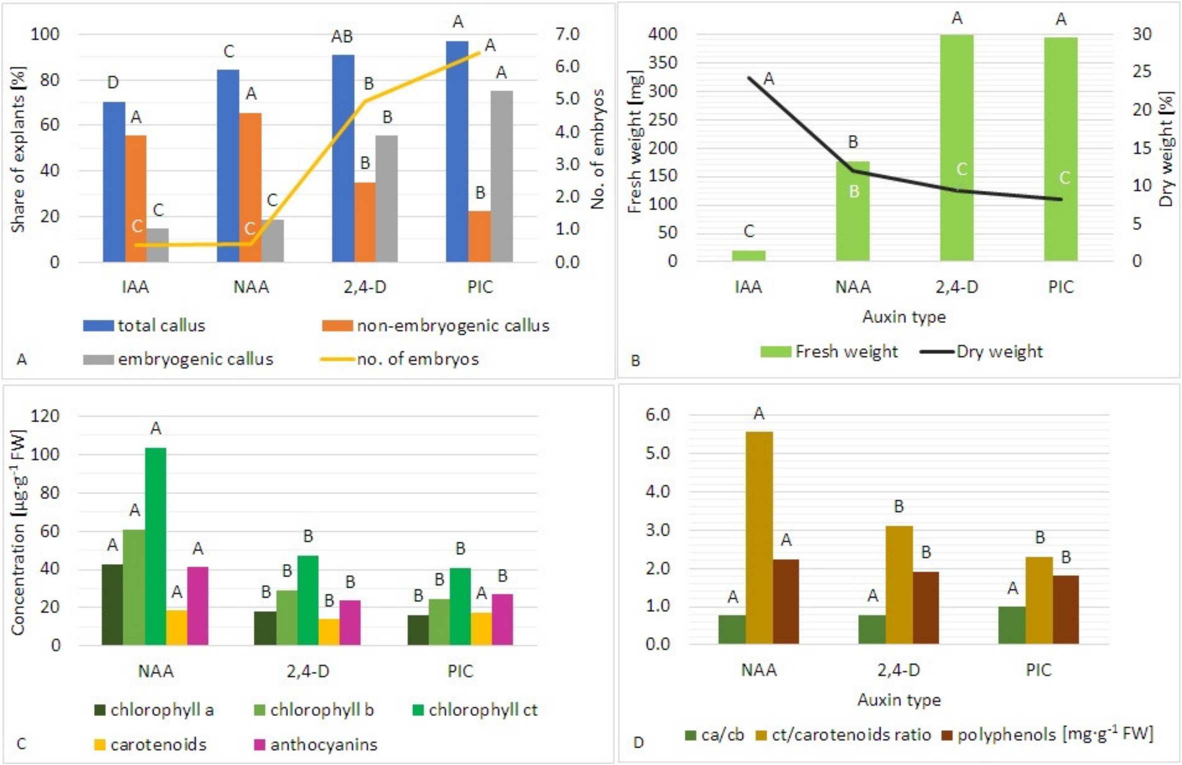

Figure S1. Influence of auxin type on the morphogenetic (**A**,**B**) and biochemical response (**C**,**D**) of bleeding heart explants, irrespective of cytokinin concentration and explant type.
